# Management of Dental School During the COVID-19 Pandemic: Application of Intervention Mapping

**DOI:** 10.3389/fpubh.2021.685678

**Published:** 2021-11-16

**Authors:** Ali Heidari, Amirfarhang Miresmaeili, Ali Poormohammadi, Saeid Bashirian, Marjaneh Meschi, Hamed Karkehabadi, Bahareh Baharmastian, Omid Aziziansoroush, Nazli Rabienejad, Samane Shirahmadi

**Affiliations:** ^1^Department of Oral and Maxillofacial Surgery, School of Dentistry, Hamadan University of Medical Sciences, Hamadan, Iran; ^2^Department of Orthodontics, School of Dentistry, Hamadan University of Medical Sciences, Hamadan, Iran; ^3^Center of Excellence for Occupational Health, Research Center for Health Sciences, School of Public Health, Hamadan University of Medical Sciences, Hamadan, Iran; ^4^Department of Health Education and Promotion, Social Determinants of Health Research Center, Hamadan University of Medical Sciences, Hamadan, Iran; ^5^Department of Community Oral Health, School of Dentistry, Hamadan University of Medical Sciences, Hamadan, Iran; ^6^Department of Endodontic, The Infection Control Committee, School of Dentistry, Hamadan University of Medical Sciences, Hamadan, Iran; ^7^Noandishan Behbood Gostar Kimia (NBGK) Company, Hamadan, Iran; ^8^Department of Periodontology, School of Dentistry Hamadan University of Medical Sciences, Hamadan, Iran; ^9^Department of Community Oral Health, School of Dentistry and Dental Research Centers, Hamadan University of Medical Sciences, Hamadan, Iran

**Keywords:** dentistry, COVID-19, infection control, intervention, evaluation, prevention

## Abstract

**Background:** Coronavirus Diesease-2019 (COVID-19) outbreak has led to the suspension of the activities of dental schools. Therefore, reorganizing clinical settings and supporting services as quickly as possible has received much attention to reopen dental schools. The present study aimed to evaluate the applicability of the Intervention Mapping (IM) approach for designing, implementing, and evaluating an intervention program to prevent and control COVID-19 in dental schools.

**Methods:** Following the IM protocol, six steps were completed in the planning and development of an intervention, targeting, and management of Dental School during the COVID-19 pandemic.

**Results:** The information obtained from the needs assessment revealed that the COVID-19 outbreak prevention was associated with the use of personal protective equipment by all target groups, infection control measures taken in the environment, preparation of the environment and equipment, changes in the treatment plan according to the COVID-19 pandemic, changing the admission process of patients, and reduction of attendance of target groups in the school are linked with. In this study, determinant factors affecting the COVID-19 prevention at the individual level were identified based on the Protection Motivation Theory (PMT). In this program, various methods, such as presentation of information, modeling role, and persuasion measures, were utilized and the practical programs included educational films and group discussions implemented.

**Conclusions:** Our findings indicated that intervention in dental environments on the basis of the IM process can develop a comprehensive and structured program in the dental school and hence can reduce the risk of the COVID-19 infection.

## Background

In recent months, the high prevalence of the new coronavirus disease-2019 (COVID-19) is considered an international public health concern (1). The virus has raised a monumental challenge to clinics and dental schools in all affected countries (2). Various treatments and control measures are performed in different healthcare systems with different economic conditions and political ideologies in every country (2).

Due to the high transmissibility of the severe acute respiratory syndrome coronavirus 2 (SARS-CoV-2), close contact between the patients and dentists, nature of dental procedures (3, 4), and difficulty in diagnosing the disease during the incubation period and in asymptomatic individuals (5, 6) all routine dental activities have been suspended in schools (7) and reduced to emergency treatments and virtual training in affected countries (8).

The activities of dental schools have been resumed by imposing some restrictions from June 21, 2020 in Iran due to the educational needs of students and the medical needs of patients. Given the vast number of patients and their close contact with students, teachers, and staff in the dental schools, some managerial, educational, and environmental interventions are necessary to be implemented for the protection of all stockholders. To reopen the schools, it is required to provide immediate educational, clinical, and support services (4).

The Centers for Disease Control and Prevention (CDC), American Dental Association (ADA), and WHO have provided some effective practical guidelines for controlling the prevalence of COVID-19 in dental settings (9–11). However, previous studies showed that the ultimate effect of health interventions depends not only on the effectiveness of the intervention but also on its accessibility in populations, proper application, the extent of providing information, awareness of the target groups, and the existence of a sanction to perform it correctly and completely (12). Inadequate implementation of effective interventions, guidelines, and policies could reduce the risk of transmission of the COVID-19 infection among patients, students, teachers, staff, and society and its spread in these environments (13, 14). Therefore, to implement the interventions and guidelines, there is a need for a systematic process contributing to the promotion and implementation of evidence-based interventions, which can identify determinants, mechanisms, and strategies for effective interventions (13). In this research, an intervention approach was introduced and described to implement a program for the prevention and control of COVID-19 in the School of Dentistry of Ibn-Sina University of Medical Sciences, Hamadan, Iran. The School of Dentistry implemented the interventions to achieve the Safety and Hygiene Scheme standard (SHS: 2020).

### Intervention Mapping

It is a protocol that helps to design multilevel health promotion interventions and selects appropriate implementation strategies (15). IM consists of six steps, and each of these steps involves a variety of tasks. Completing tasks at each stage serves as a guide for the next steps. Completing all steps acts as a work plan for designing, implementing, and evaluating interventions, that is based on theoretical, experimental, and practical information. The steps are as follows:

Step 1: Conducting needs assessment

Step 2: Specifying change objectives

Step 3: Selecting theory-based methods and practical strategies

Step 4: Developing program components

Step 5: Specifying implementation

Step 6: Generating evaluation plan (15).

Steps 1–4 focus on the presentation of multilevel interventions for improving health behaviors and environmental conditions.

## Methods

### Step 1: Conducting Needs Assessment

In the first step of IM, planners evaluate assets and needs.

At this stage, all representatives, such as executives and those who are responsible for protecting the intervention, were involved in the program to determine required measures for implementing the program and identifying determinants (obstacles and facilitators). To involve patients in the program, a committee called the COVID-19 Prevention Committee at the School of Dentistry (CPDS) was formed on May 21, 2020. The members of this committee consist of the representatives of the students, the chairman of the infection control committee of the school, representative of the university teachers, staff representative, manager of the nursing office, patient representative, an IT official of the school, representative of the department of financial and administrative affairs of the school, the head of public affairs, the dean of the school, and some SHS2020 standard company's representatives. The recognized stakeholders were in both individual and environmental levels. The team manager (Head of the School) determined the responsibilities of each team member and the decision-making structure. During this session, the members of the three teams, namely, education, infection control, and logistics, were separately assigned for performing relevant activities.

To assign all actors, barriers, and potential facilitators of implementing the intervention, it was necessary to conduct a needs assessment. For this reason, the brainstorming method was used in CPDS, and the results of the previous regular evaluation that have already been conducted in the school were applied. Moreover, the environmental conditions were observed and evaluated.

At this point, social, epidemiological, and behavioral-environmental analyses were performed. The needs assessment of the present study was focused on the school as the target group, such as teachers, students, staff (medical and administrative), and patients. This entailed a review of literature on determinants and factors associated with the prevalence of COVID-19 in dental settings and the role of behaviorally and environmentally predetermined risk factors in the rise of the prevalence of COVID-19 and its determinants. The committee used the experimental data, theoretical references, and collected new data (through focus group interviewing with students, patients, and infection control members of the school) to prepare a list of behavioral and environmental risk factors affecting the spread of COVID-19 and its determinants. In this phase, recognition of the school capabilities was achieved.

### Step 2: Specifying Change Objective

At this stage, CPDS identified the expected changes in the behavioral and environmental factors, which were already determined in the needs assessment stage. The prioritization of behavioral and environmental consequences was performed according to their importance and variability by literature review and the consensus between the members of the CPDS committee. Afterward, functional objectives were determined for each behavioral and environmental consequence and their determinants, and a matrix of change objectives was plotted for each program level. We used brainstorming, a review of literature, and constructs of health promotion theories to identify the determinants.

### Step 3: Selecting Theory-Based Methods and Practical Strategies

In this step of CPDS, the brainstorming method and literature review were used to identify the appropriate methods and strategies affecting the determinants in step 2. During this process, a summary of the theoretical methods proposed by Bartholomew et al. was applied (15). Special efforts were made for searching and selecting existing strategies, which are consistent with the theoretical methods and specific objectives of the intervention.

### Step 4: Developing Program Components

In this step, the intervention content was proposed by the CPDS team, and the protocols of implementation, activities, and training materials were produced. The cultural adaptation of these programs was conducted through consultation with the program stakeholders. Then, the scope, sequence, communication channels, themes, and a list of required materials were provided and documented. Meanwhile, a review of the existing training materials was performed to determine whether the training materials have already been consistent with the objectives of this program or not. Afterward, the required training materials were produced and pre-tested.

### Step 5: Specifying Implementation

At this step, the potential adopters and implementers of the program were identified. A review of the transplantation system (CPDS) was also performed. Then, the functional objectives of adoption, implementation (in terms of loyalty, comprehensiveness, and dose), and program survival were identified. In the next stage, the determinants of adoption, implementation, and survival of the program were determined using the brainstorming method and literature review. Based on the importance and variability, the prioritization of the change objectives was conducted. Then, appropriate methods and strategies affecting the determinants of adoption, implementation, and survival of the program were selected, and the intervention was designed and developed to use the program.

### Step 6: Generating Evaluation Pan

In the final stage of IM, a plan was provided by the planners to evaluate the effectiveness and quality of the intervention (15). In the implemented program evaluation, the content of the questions was designed based on the information collected from the needs assessment and other previous steps of the IM.

A checklist was designed for the evaluation of behavioral and environmental factors, performance objectives, determinants, and change objectives. A checklist was also designed for the process evaluation. Evaluation indicators and criteria were determined, and the evaluation plan was documented. To evaluate the program, the context, input, process, and product (CIPP) evaluation checklist was utilized (16). The evaluation process was prepared by the CPDS team as soon as the agreement was reached on the content of the intervention.

### Ethical Consideration

The protocol of this study was approved by the Ethics Committee of the Hamadan University of Medical Sciences (approval code: IR.UMSHA.REC.1399.416). The questionnaire was anonymous, and informed consent was obtained from the participants in the survey. Participation in the study was voluntary.

## Results

### Step 1: Conducting Needs Assessment

As of May 21, 2020, a total of 129,341 cases with COVID-19 have been laboratory confirmed and 7,249 deaths diagnosed (11); and these numbers are rising. According to the Occupational Safety and Health Administration (OSHA) report, the healthcare workers who are exposed to aerosols generated during the examination or treatment of patients are at high occupational risk for COVID-19 (17, 18). Due to the unique features of dental procedures that generate a large number of droplets and particles suspended in the air. Moreover, due to the exposure of dental staff to blood, body fluids, saliva, and microorganisms in the mouth, nose, and respiratory secretions, the possibility of cross-infection between dentists and patients is very high (19). Therefore, dentistry is one of the most important occupations in terms of COVID-19 transmission and involvement. At this stage, the recipients of the program were identified, such as students, professors, medical staff, administrative staff, and patients. The literature review showed that the use of personal protective equipment by all target groups, performing infection control measures in the dental operatory area, preparing the environment and equipment, changing the way of queuing and admission of patients, reducing capacity and attendance of patients to reduce population density per unit area, and reducing the exposure time of the target groups in the departments are associated with the prevention of the spread of the COVID-19 disease (20, 21).

One of the key points in the control of infectious disease epidemics is designing interventions to emphasize the simultaneous increase of perceived fear and threat and perceived efficacy (such as, the effectiveness of the proposed strategies) for disease prevention and self-efficacy. Therefore, Protection Motivation Theory (PMT) was used to better understand the performance of preventive behaviors at an individual level (Table 1).

**Table 1:** Identifying determinants being affected by COVID-19 in the school of dentistry.

	**Additions from new data**	**Additions from theory**	**Additions from literature**
**Personal determinants**			
Students and teachers	Knowledge of the latest treatment instructions in COVID-19 conditions Fear of getting COVID-19 Fear of lack of personal protective equipment in the school Knowledge of how to use personal protective equipment Knowledge of how to dispose of infectious waste hygienically Fear of lack of disinfectants in college	Intention to use personal protective equipment (Theory of Planned Behavior) Perceived barriers to use personal protective equipment (Health Belief Model) Perceived susceptibility (Health Belief Model) Self-efficacy (Social Cognitive Theory) Perceived severity (Health Belief Model) Response efficacy (Protection Motivation Theory)	Proper washing of instruments Proper disinfection of instruments Proper disinfection of equipment Proper environmental disinfection Proper disinfection of templates, casts and apps Wear personal protective equipment Failure to perform elective treatments Wash your hands regularly with soap and water or antiseptic Fear of getting COVID-19 High perceived threat rate High perceived efficiency Observing social distancing from teachers and other students
Staff and patients	Knowledge of the latest COVID-19 prevention guidelines Fear of getting COVID-19 Use of personal protective equipment (mask) Fear of COVID-19 complications	Intention to use personal protective equipment (Theory of Planned Behavior) Perceived barriers to use personal protective equipment (Health Belief Model) Perceived susceptibility (Health Belief Model) Self-efficacy (Social Cognitive Theory) Perceived severity (Health Belief Model) Response efficacy (protection motivation theory)	Proper disinfection of the environment Regular hand washing with soap and water or antiseptic Fear of getting COVID-19 Observing social distancing from patients Non-use of public stuff such as pens, magazines, telephones, etc., Arranging a scheduled appointment
**Environmental determinants**			
Students and teachers	Shared buffet and self-service Dormitories with limited physical space SMALL physical spaces of changing rooms Small physical space of some medical wards Small physical space of some teachers' rooms.		Strong air conditioning for waiting rooms and treatment wards Strong saliva suction for each unit Creating isolated units Observing safe distance of the units Anti-retraction hand-pieces Suitable disinfectants Virtual teaching Installation of Picture Archiving and Communication Systems software (PACS)
Workers	Small physical space for some workers		Reduction of staff's attendance in the school
Patients	Lack of proper physical space for patient's waiting Patient entry and exit body control		Reduction of patient's attendance in the school Changing patient's queuing and admitting method Teledentistry Phone or online screening of patients

A high commitment of management to implement the intervention, high level of staff education, access to new device/equipment, sufficient budget, sufficient workforce to implement the intervention, and presence of qualified professors in the field of infection control were among the strengths of the faculty.

### Step 2: Specifying Change Objective

Based on the information obtained from the needs assessment stage, two objectives of the program (at the individual and environmental levels) were considered to prevent the spread of the COVID-19. For the target group, such as students and professors, the program was defined at the individual level as the use of appropriate personal protective equipment and suitable treatment measures to prevent the spread of COVID-19 (such as reducing exposure time and social distance).

In the target group of administrative staff and patients, the implementation of the preventive behaviors to prevent the transmission and spread of COVID-19 was considered as the program objective at the individual level.

Due to the important role of the medical staff and service personnel in infection control, the use of appropriate personal protective equipment and precise infection control measures among them was considered at the individual level in addition to the other staff.

In addition, the objectives of the program for environmental factors were to prepare the environment and equipment, change the system of queuing and admission of patients, and reduce the capacity of target groups in the faculty. According to the program objectives, performance objectives were defined at the individual level for students, professors, administrative staff, medical staff, and patients, and the performance objectives were defined at the environmental level for providers (faculty officials).

After identifying the functional objectives, the important and changeable determinants of these objectives were selected for each level based on the results of the IM phase. For the individual level, PMT was selected as a framework for describing factors in relation to preventive behaviors of COVID-19 (22). Another important determinant was knowledge about the latest COVID-19 prevention guidelines in dental settings. Table 2 presents an example of this matrix.

**Table 2:** The matrix of change objectives for teacher and students.

**Personal determinants**								**External determinants**
**Performance objectives**	**Knowledge**	**Perceived susceptibility**	**Perceived severity**	**Self-efficacy**	**Response efficacy**	**Intention**	**Cost**	**Availability and accessibility**
**Behavior 1: Teacher and students use personal protective equipment**								
PO1.1.- Professors and students should be familiar with the personal protective equipment required at the time of COVID.	K1.1.a- They explain what personal protective equipment should be used by dentists during the COVID period according to the latest published guidelines. K1.1.b- Teachers and students to name the personal protective equipment required for the administrative, pre-clinical and clinical departments.	PSU (1.1–1.4)- teachers and students find themselves at risk for COVID 19.	PS (1.1–1.4). a- Teachers consider COVID disease deadly. PS (2.1–2.4). b- Teachers are concerned about their students' complications from COVID disease. PS (2.1–2.4). c- Teachers are concerned about COVID-19 disease affecting family and relatives through themselves.	SE1.1- Students and teacher believe that they can search, receive and study the latest published information on personal protective equipment needed by dentists during the COVID period.	RE1.1- Students and teacher believe that knowing the latest instructions on personal protective equipment needed during the COVID period will prevent them from contracting the disease.	I1.1- Dental teachers and students intend to receive and read the latest instructions related to personal protective equipment every 48 h.	C1.1- Students and teacher describe searching, receiving, and reading the latest instructions as time consuming.	AA1.1- The teacher should provide the latest instructions to teachers and students.
PO1.2- Professors and students receive their personal protective equipment from the school.	K1.2- Students explain which department they should go to get personal protective equipment.			SE1.2- Students believe that they can request the personal protective equipment they need from the relevant authorities.				
PO1.3- Professors and students wear personal protective equipment needed for each ward.	K1.3.a- Students and professors should state the benefits of using personal protective equipment. K3.3.b- Students and professors explain how to wear personal protective equipment properly. K3.3.c- Students and faculty to explain how to properly remove personal protective equipment. K1.3.d- Students and teachers demonstrate how to wash their hands properly. K1.3.e- Students and professors state the order of wearing personal protective equipment. K1.3.f- Students and teachers state the order of removing personal protective equipment.			SE1.3.a- Students and teachers believe that they can wear personal protective equipment correctly.SE1.3.b- Students and professors believe that they can remove personal protective equipment correctly.SE1.3.c- Students and professors believe that they can follow the order of wearing personal protective equipment.SE1.3.d- Students and teachers believe that they can wash their hands properly before putting on and taking off personal protective equipment.SE1.3.e- Students and teachers believe that they can follow the order of removing personal protective equipment.SE1.3.f- Students and teachers believe that they can properly use personal protective equipment related to clinical and pre-clinical wards.	RE1.3.a- Students and teachers believe that following the tips for wearing the personal protective equipment needed during the COVID period will prevent them from contracting the disease. RE1.3.b- Students and teachers believe that following the tips for properly removing the personal protective equipment needed during the COVID period will prevent them from contracting the disease. RE1.3.c- Students and teachers believe that the proper use of personal protective equipment associated with clinical and pre-clinical wards will prevent them from contracting COVID-19 disease. RE1.3.d- Students and teachers believe that hand washing before wearing and after removing personal protective equipment prevents them from developing COVID-19 disease	I1.3.a- Dental professors and students intend to use personal protective equipment for the entire period of the COVID-19 epidemic.I1.3.b- Dental professors and students intend to wear personal protective equipment correctly during the entire period of the COVID epidemic.I1.3.c- Dental professors and students intend to remove personal protective equipment in the correct manner during the entire period of the COVID-19 epidemic.I1.3.d- Students and teachers plan to wash their hands with soap and water before wearing and removing personal protective equipment throughout the COVID-19 epidemic.	C1.3.a- Students and teachers describe wearing personal protective equipment as time consuming. C1.3.b- Students and teachers describe wearing personal protective equipment as annoying.	AA1.3.a- The school provides teachers and students with personal protective equipment suitable for office, pre-clinical and clinical environments.AA1.3.b- The school should provide students and teachers with liquid soap and hand sanitizers.
PO1.4- Professors should supervise how students wear personal protective equipment.	K1.4.a- Teachers should explain how to complete the personal protective equipment evaluation checklist.			SE1.4.a- Teachers believe that they can monitor how their students wear personal protective equipment	RE1.4- Professors believe that monitoring how students wear personal protective equipment prevents them, their students, and their patients from developing COVID-19 disease.	I1.4- Professors intend to monitor how their students wear personal protective equipment throughout the COVID-19 epidemic.	C1.4- Teachers describe how time consuming it is for students to wear personal protective equipment.	AA1.4- The school provide teachers with checklists related to the supervision of students' personal protective equipment.
**Behavior 2: Teacher and students perform infection control actions**								
PO2.1- Wash and disinfect hands before and after contact with the patient, instruments and surfaces properly	K2.1.a- Students and professors should show how to wash their hands properly. K2.1.b- Students and professors should describe the benefits of hand washing before and after contact with the patient, instruments and surfaces in the correct way.	PSU (2.1–2.4)- Professors and students find themselves at risk for COVID 19.	PS (2.1–2.4). a- Professors consider COVID disease deadly. PS (2.1–2.4). b- Professors should be concerned about their students' complications from COVID disease. PS (2.1–2.4). c- Professors should be concerned about COVID-19 disease affecting family and relatives through themselves.	SE2.1- Students and teachers believe that they can wash their hands, instruments and surfaces properly before and after contact with the patient.	RE2.1.a- Students and teachers believe that hand washing before and after contact with the patient, instruments and surfaces will prevent them from developing COVID-19 disease. RE2.1.b- Students and teachers believe that hand washing before and after contact with the patient, instruments and surfaces will prevent their dental patients from developing COVID-19 disease.	I2.1- Students and teachers intend to wash their hands, instruments, and surfaces with soap and water before and after contact with the patient throughout the COVID epidemic.	C2.1- Teachers and students describe hand washing before and after contact with patient, instruments and surfaces as time consuming.	AA2.1- The School provides students and teachers with liquid soap and hand sanitizers.
PO2.2- Use of sterile disposable packs for each patient (disposable covers of the unit, lamp handle, suction cup, weather power, disposable cup, apron, turbine and angle).	K2.2- Students and professors should explain the reason for using disposable packs for each patient.			SE2.2- Students and faculty believe that they can use sterile disposable packs for any patient.	RE2.2.a- Students and faculty believe that the use of sterile disposable packs will prevent them from developing COVID-19 disease. RE2.2.b- Students and faculty should believe that the use of sterile disposable packs prevents their dental patients from developing COVID-19 disease.	I2.2 Students and teachers intend to use sterile disposable packs for each patient throughout the COVID-19 epidemic.	C2.2- Professors and students describe the use of sterile disposable packs as time consuming.	AA2.2- The Faculty should provide students and professors with sterile disposable packs for each patient.
PO2.3- Sanitary disposal of infectious waste (personal protective equipment and waste)	K2.3.a- Students and professors should explain how to dispose of disposable personal protective equipment. K2.3.b- Students and professors should explain how to dispose of infectious waste. K2.3.c- Students and professors should explain how to dispose of sharp and keen instruments. K2.3.d- Students and teachers should describe the characteristics of the bin for infectious and non-infectious waste.			SE2.3.a- Students and professors believe that they can dispose of their personal protective equipment in a hygienic way after use.SE2.3.b- Professors and students believe that they can dispose of infectious waste in a hygienic way.SE2.3.c- Students and professors believe that they can dispose of sharp and keen instruments in a safe and hygienic way.	RE2.3.a- Students and faculty believe that hygienic disposal of personal protective equipment prevents them and their patients from developing COVID-19 disease. RE2.3.b- Students and faculty believe that hygienic disposal of infectious waste prevents them and their patients from developing COVID-19 disease. RE2.3.c- Students and professors should believe that hygienic and safe disposal of sharp objects prevents them and their patients from developing COVID-19 disease.	I2.3- Students and professors intend to dispose of infectious wastes generated during the treatment of patients in a hygienic way during the entire period of the COVID-19 epidemic.		AA2.3.a- The school shall provide a recycled bin with the appropriate number for each department.AA2.3.b- The school shall provide the yellow garbage bag for infectious waste and the black garbage bag for non-infectious waste as required for each clinical ward and pre-clinical ward.
PO2.4- Washing and sterilizing reusable personal protective equipment	K2.4.a- Students and professors should explain how to wash the instruments used. K2.4.b- Students and professors should name disinfectants suitable for disinfection of instruments and equipment. K2.4.c- Students and professors should explain the correct way to prepare disinfectants. K2.4.d- Students and professors should name personal protective equipment that should be disinfected after the examination of each patient. K2.4.e-Students name the person or place who or where they must hand over their instruments after washing.			SE2.4.a- Students should believe that they can wash the instruments used properly.SE2.4.b- Students and teachers should believe that they can properly disinfect personal protective equipment that can be disinfected.SE2.4.c- Students and teachers believe that they can prepare the disinfectant solution correctly.	RE2.4- Students and teachers should believe that proper washing and disinfection of instruments and equipment will prevent them and their patients from developing COVID-19 disease.	I2.4- Students and professors intend to wash and disinfect their instruments and equipment properly after use during the entire period of the COVID epidemic.		AA24.a- The school should build a suitable place next to the unit for washing instruments after use.AA2.4.b- School should provide students and teachers with appropriate disinfectants.
**Behavior 3: Appropriate treatment actions for COVID-19 period**								
PO3.1- Providing less aerosol treatment plan and less saliva contact	K3.1- Students and professor explain treatment plans that generate fewer aerosols in connection with each type of treatment.	PSU (3.1–3.4)- Students and professors find themselves at risk for COVID 19.	PS (3.1–3.4). a- Professors consider COVID disease deadly. PS (3.1–3.4). b- Professors are concerned about their students' complications from COVID disease. PS (3.1–3.4). c- Professors are concerned about COVID-19 disease affecting family and relatives through themselves.	SE3.1- Students and teachers believe they can use treatment plans that aerosols are less generated in treating patients.	RE3.1- Students and teachers believe that using treatment plans that generate fewer aerosols will prevent them and their patients from developing COVID-19 disease.	I3.1- Students and teachers intend to use aerosol-less treatment plans throughout the duration of the COVID-19 epidemic.	C3.1- Professors and students describe the use of less aerosol treatment plan as time consuming.	
PO3.2- Practicing 4-handed dentistry	K3.2.a- Students and professors should explain four-handed dentistry. K3.2.b- Students and teachers should discuss the benefits of using four-handed dentistry during the corona.			SE3.2 Students and teachers believe that they can use four-handed dentistry while treating patients.	RE3.2- Students and teachers believe that the use of 4-handed dentistry prevents them and their patients from developing COVID-19 disease.	I3.2- Students and faculty intend to use four-handed dentistry throughout the COVID-19 epidemic.		
PO3.3- Using rubber dam	K3.3.a- Students and professors should explain how to use rubber dam K3.3.b- Students and teachers should discuss the benefits of using rubber dam. K3.3.c- Students and professors should name the treatments in which rubber dam must be used during the treatments.			SE3.3- Students and teachers believe that they can use rubber dam while treating patients.	RE3.3- Students and teachers believe that the use of rubber dam prevents them and their patients from developing COVID-19 disease.	I3.3- Students and teachers intend to use rubber dam for the entire duration of the COVID-19 epidemic.	C3.3.a- Professors and students describe the use of rubber dam as time consuming. C3.3.b- Professors and students describe the use of rubber dam as difficult.	AA3.3- The school should provide students and teachers with rubber dam.
PO3.4- Re-screening patients with respect to COVID	K3.4.a- Students and professors know the signs of COVID. K3.4.b- Students and teachers should identify suspected cases of COVID. K3.4.c- State the risk factors associated with COVID. K3.4.e- Students and professors should explain how to deal with a disease that manifests the symptoms of COVID during examination and treatment.			SE3.4.a- Students and faculty believe that they can correctly identify the symptoms and risk factors of COVID.SE3.4.b- Students and teachers believe that they can function properly when dealing with a patient suspected of having COVID.	RE3.4- Students and faculty believe that proper screening of patients prevents them and their patients from developing COVID-19 disease.	I3.4- Students and teachers intend to screen patients throughout the COVID-19 epidemic.	C3.4. Professors and students describe patient screening as time consuming.	AA3.4- The school should provide students and professors with screening forms.
PO3.5- Treatment of emergency patients	K3.5.a- Students and professors name the types of emergency cases. K3.5.b- Students and professors should provide examples of emergency cases. K3.5.c- Students and teachers should explain the tree of decision making on emergency dental services.			SE35.a- Students and faculty believe that they can correctly identify patients' medical needs.	RE3.5- Students and teachers believe that the lack of elective treatments will prevent them and their patients from developing COVID-19 disease.	I3.4- Students and professors intend to refrain from treating elective patients during the entire period of the COVID-19 epidemic.		
**Behavior 4: Observance of social distance**								
PO4.1- Observance of social distancing from patients (at a time other than during treatment and examination), other students, professors and other faculty members	K4.1.a- Students and teachers should state the amount of social distancing they should observe. K4.1.b- Students and teachers should state the benefits of not gathering on the campus.	PSU 4.1- Professors and students find themselves at risk for COVID 19.	PS 4.1.a- Professors consider COVID disease deadly. PS (2.1–2.4). b-Professors should be concerned about their students' complications from COVID disease. PS (2.1–2.4). c- Professors should be concerned	SE 4.1- Students and teachers believe that they can maintain a distance of 1.5–2 m with their patients (at a time other than treatment) students, teachers, and other staff.	RE4.1.a- Students and professors believe that the use of four-handed dentistry prevents them and their patients from developing COVID-19 diseases. Students and professors believe that maintaining social distancing can prevent them and their patients from developing COVID-19 RE4.1.b- Students and teachers believe that avoiding gatherings can prevent them from developing COVID 19.	I4.1- Students and professors intend to maintain a distance of 1.5–2 m from their colleagues and patients during the entire period of the COVID-19 epidemic.		
		about COVID-19 disease affecting family and relatives through themselves.						

Finally, for providers (school's officials) at the environmental level, the knowledge of the latest COVID-19 prevention guidelines in dental settings, barriers, attitudes, and outcome expectations were chosen as determinants. Next, the matrix of change objectives, which is derived from the intersection of performance objectives with determinants, was formed for each level of intervention (Table 3).

**Table 3:** The matrix of change objectives for providers.

**Performance objectives**	**Determinants**			
	**Knowledge**	**Outcome expectations**	**Barriers**	**Attitude**
**Environmental change: established safety and hygiene scheme (SHS2020)**				
PO1.1- School's officials should seek funding for a safety and health plan.PO1.2- Officials should buy the necessary materials, instruments and equipment at a reasonable price.	K1.1.a- They should describe the process of funding.K1.1.b- they should list the places where materials and equipment are sold at reasonable prices.K1.1.c- they should prepare a list of required materials, instruments and equipment.	OE1.1- Officials should expect funding for a health and safety plan can prevent the spread of COVID disease at the school.OE1.1- Officials should expect that the purchase of materials, equipment and supplies will prevent the spread of COVID disease in the faculty.	B1.1.a- Officials should state that the funding process is required during the administrative and paperwork process.B1.1.b- Officials plan how to meet the demand for money when there is insufficient funding.B1.1.b- Officials plan how to deal with the purchase of instruments and equipment when they are not available.	A1.1- Officials believe that the establishing a safety and health plan is an important part of the care of the target groups covered by them.
PO2.1- They should plan the installation of new equipment, isolation of units, improvement of air conditioning systemPO2.2- They should launch the online queuing and registration system.PO2.3- They should plan to inform the public of the new method of queuing and admitting in the schoolPO2.4- They should plan to reduce the presence of target groups to one third of the capacity (by using telephone or online appointment system, teledentistry, implementation of Virtual teaching, phone or online screening of patients and installation of PACS software).PO2.5- They should plan to stick up educational poster regarding COVID and signs of social distancing.PO2.6- Separation of patients' entry and exit doors from staff	K2.1.a- Specialists recognize the installation of equipment, isolation of units and examination of ventilation system and launch of telephone queuing system.K2.1.b- Identify virtual teaching platform specialists and PACS software.K2.1.c- Determine the resident students to provide teledentistry.	OE.2.1.a- They should expect that the installation of new equipment, isolation of units, improvement of the air conditioning system will prevent the outbreak of Corona disease in the school.OE.2.1.b- They should expect the launch of a telephone and online queuing system can prevent the spread of Corona disease in the school.OE.2.1.c- They should expect the launch of teledentistry can reduce the presence of students and teacher in the school.OE.2.1.d- They should expect the launch of a virtual teaching can reduce the presence of students and teacher in the school.OE.2.1.e- they should expect the launch of PACS software can reduce the presence of students and teacher in the school.OE.2.1.f- They should expect informing the general public about the manner of queuing and telephone and internet admitting can prevent patient congestion in the school.OE.2.1.g- They should expect sticking up of educational posters related to COVID and signs of social distancing can prevent the spread of corona disease in the school.OE.2.1.h- They should expect separation of patient entry and exit doors from staff can prevent the spread of coronary disease in the school.	B2.1.a- Officials should state that the installation of new equipment, isolation of units, improvement of the air conditioning system requires administrative and paperwork procedures.B2.1.b- Officials should state that setting up an online and telephone queuing system requires manpower and equipment.B2.1.c- Officials should state that setting up an online and telephone appointment system requires manpower and equipment.B2.1.d- Officials should state that reducing the presence of target groups to one third of capacity and separating the entrance and exit doors of patients from staff require administrative and paperwork procedures.B2.1.e- Officials should state that the separation of patients' entry and exit doors from staff required manpower.B2.1.f- Officials should state that the launch of teledentistry required manpower and E-Infrastructure.B2.1.g- Officials should state that the launch of Virtual teaching requires manpower and E-Infrastructure.B2.1.h- Officials say that setting up virtual teaching requires holding training courses to familiarize students and teachers with classes virtually.	A2.1- Officials should believe that establishing a safety and health plan is an important part of the care of the target groups covered by them.
PO3.1-Hold training courses for students, faculty and staff on COVID- 19 and ways to prevent it.		OE.3.1- They should expect that holding COVID-19 training courses for students, faculty and staff will increase their COVID-19 awareness and ways to prevent it.	3.1.a- Officials should state that holding COVID-19 training courses for students, faculty and staff requires Internet infrastructure.B3.1.b- Officials should state that holding COVID-19 training courses for students, faculty and staff requires large halls.	A3.1- The belief that holding COVID-19 training courses for students, teachers, and staff is an important part of the school's role in preventing the spread of Corona in society.

These performance objectives have been developed based on the protocols of the WHO, CDC, and the Ministry of Health and Medical Education of Iran (9–11).

To reduce the presence of target groups in the school, contact-less treatment and remote learning were considered. This intervention was performed by using a telephone or online appointment system, teledentistry, implementation of virtual teaching, phone or online screening of patients, and installation of the Picture Archiving and Communication Systems software (PACS). The patient screening was conducted through a telephone call or appointment system so that before making an appointment, the patient was asked to complete the COVD-19 screening. After completing the screening form, if he/she was not identified as a high-risk case, was referred to oral diseases resident to do teledentistry. At this level, patients would express their symptoms and problems, and then if necessary, would send a photograph of their mouth to the oral diseases resident. The resident determines whether the person needs essential or urgent treatment or not based on the patient history and the provided photograph. An appointment was considered for the patients who need special treatments.

Virtual teaching was considered for theoretical courses. Adobe connect platform was considered for theoretical courses and webinar.

The installation of PACS software, which reduces paper records, increases the speed and accuracy of diagnoses and subsequently reduces the patient waiting time and their contact with staff, students, and faculty in the department. The PACS software could increase the speed and accuracy of diagnoses and subsequently reduce the patient waiting time and their contact with staff, students and faculty in the department.

### Step 3: Selecting Theory-Based Methods and Practical Strategies

At this stage, all the change objectives were organized by the relevant determinants, and the methods of each category of the objectives were identified through the brainstorming method. Afterward, a theoretical and experimental review was performed to identify the appropriate methods and strategies for changing determinants.

The strategy planning team determined the strategies in two ways, such as (1) brainstorming and documenting ideas about strategies, materials, and communication channels during the planning process; and (2) thinking about selected methods and deciding on the specific strategies that can make them practical. Table 4 represents the selected methods and practical applications for changing the factors determined at each level of the intervention.

**Table 4:** Overview of the selected theoretical methods and practical strategies used in the intervention.

	**Change Objective**	**Method**	**Strategy**
Individual	Knowledge	Active learning/providing information/Feedback/Persuasive communication	Webinars Providing written and verbal Information (lecture, pamphlet), question and answer between the educators and the employee Video presentation - Role playing
	Perceived Susceptibility, Perceived Severity,	Fear arousal Personalize risk	Professors should state the high morbidity rates of the disease, all target groups' exposure to risks, the high risks of dental environments, and discuss these issues about the risks of coronavirus.
	Self-Efficacy	Guided practice, enactment, verbal persuasion, modeling, demonstration	Professors provide students with feedback on how to use personal protective equipment Prevent the entry of students and professors who do not wear personal protective equipment proportionate to the administrative, pre-clinical and clinical departments/ Prevent the entry of the students and professors who do not properly follow infection control Professors and students who use less-aerosol treatments, patient screening, and four-handed dentistry and observe social distancing, and correctly identify patients' treatment needs should be encouraged. Students' infection control should be monitored and they are given feedback. Professors as role model practice the proper way of using personal protective equipment, infection control, and hygienic disposal of waste, social distancing.
	Response efficacy, cost	Shifting perspective/belief selection	Discussion
	Intention	Active processing of information	Discussion
	Availability and accessibility	Facilitation	The school's faculty works with the university president to change policies. Officials facilitate purchasing orders and maintenance requests at the school level
Environmental	Knowledge	Information delivery	- Webinars, virtual teaching
	Outcome expectations, barriers, attitude	Facilitating conditions, Discussion	Discussion

### Step 4: Developing Program Components

In this step, a final intervention framework was produced for implementation. Based on the needs assessment data and the additional focus group discussion with the target group, to collect the data regarding intervention material preferences, the planning team provided a list of intervention materials and activities. In practical, in this intervention, the COVID-19 instructions recommended by Iran's Ministry of Health and the instructions in relation to infection control were considered (9).

A three-part educational video entitled “Providing Dental Services in COVID-19 Conditions” was also provided by the Hamadan University of Medical Sciences based on the latest instructions of the Ministry of Health. Photos, posters, and signs were received and prepared from the Ministry of Health website. The video clip was displayed for residents.

**Figure 1:**
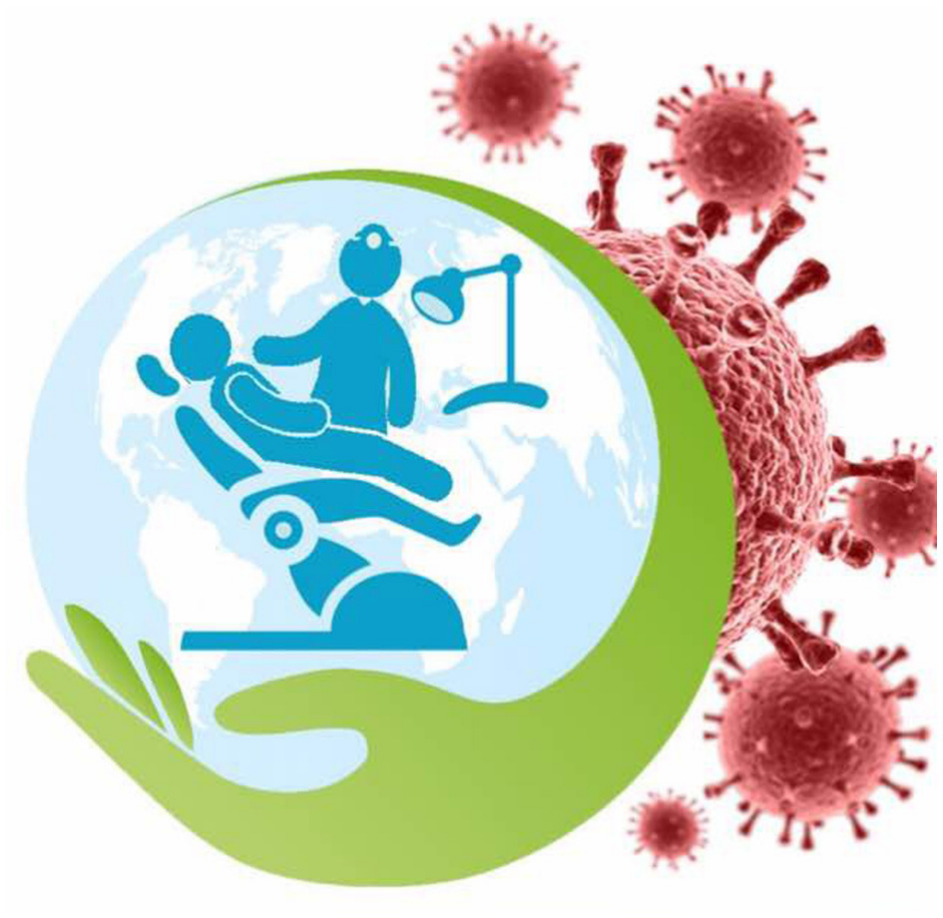
Program logo.

The intervention was implemented at the faculty through the development of intervention modules. Table 5 presents an overview of the interventions and their related modules. The intervention at the faculty level consisted of five intervention modules (modules 1–5). A logo was designed for the program, which expresses an important health message to faculty stakeholders (Figure 1). During the formative phase, positive recommendations were received regarding the designed logo.

**Table 5:** Program scope and sequence.

**Timing**	**Module 1 Environmental change**	**Module 2Educating of teachers and students**	**Module 3 Educating of medical staff**	**Module 4Educating of Administrative staff**	**Module 5 Educating of patients**
Week 1	-Clean and repair exhaust fans in the school -Installing net for windows -Installing canopies for windows - Installing anti-retraction hand-piece Collecting shared public items such as school's water coolers, magazines from the library section, prayer seals and shared pens -Installing eyed water valves in sanitary services - Putting a trash can with a lid with a yellow plastic bag for all medical units Providing necessary infrastructure for Virtual teaching programs	The first training session for professors and studentsThe instructor provided information about the disease and its symptoms and the importance of ways to prevent it, the high incidence and mortality of the disease, the high risk of dental environments and dental profession. Besides, the question-answer strategy was used to get better understanding of these issues. Some discussion was held about the issued to increase the perceived threat.The second training session for professors and students:The trainer delivered a speech about the diagnosis of essential and emergency treatments and the treatment projects that generate fewer aerosols, and a discussion was held about the issues to increase the perceived efficiency. The students and professors were offered tips on the consequences of not following the above points.			
Week 2	- Setting up a telephone and online appointment and reception system -Setting up teledentistry Setting up PACS - Informing the public about appointments and telephone and internet admissions by installing billboards in the university - Printing and duplicating screening forms	The third training session for professors and students:The trainer explained about the correct use of personal protective equipment, personal protective equipment required by each department, infection control (disinfection of instruments, equipment and environment), and sanitary disposal of infectious waste. And discussions were held about the issues to increase perceived efficiency.The fourth training session for professors and students:Training on the maintaining and following of social distancing and action in emergencies through role playing. The students and professors were offered advice on the failure to observe the above points.			
Week 3	- Isolation of 4 units in each treatment unit for examination and treatment - Installation of UV device in medical wards - arranging a schedule for the presence of professors, staff and students - Establishing laws regarding the wearing of personal protective equipment, infection control and sanitary disposal of infectious waste and the		The first training session for medical staff: The instructor provided information about the disease and its symptoms and the importance of ways to prevent it, the high incidence and mortality of the disease, the high risk of dental environments and dental profession. Besides, the question-answer strategy was used to get better understanding of these issues. Some discussion was held about the issued to		-Activation of education department for patients: patients should be offered information about the presence of patients and requirements that must be observed during their entry and attendance in the school The information was given to them when
	obligation to comply with the rules by students, medical staff and administrative staff by considering rewards and punishments to affect self-efficacy		increase the perceived threat. The second training session for medical staff: The trainer delivered a speech about the correct use of personal protective equipment, personal protective equipment required by each department, infection control (disinfection of instruments, equipment and environment), and sanitary disposal of infectious waste. Some discussions were held about the issues raised to increase the perceived efficiency. Training on the maintaining and importance of observing social distancing and action in emergencies through role playing. The medical staff was advised of the consequences of not following the said points.		they contacted the school for an appointment. The patients were advised of the failure to follow the foregoing points. Patient training was also achieved through posters and related marks of COVID.
Week 4	Installing spacing signs in waiting halls and office environments, library and prayer hall - Installing notification boards - sticking up educational posters - Separating entry and exit doors for staff and patients - deploying disinfection ponds			The first session for administrative staff:The instructor provided information about the disease and its symptoms and the importance of ways to prevent it, the high incidence and mortality of the disease, the high risk of dental environments. Besides, the question-answer strategy was used to get better understanding of these issues. Some discussion was held about the issued to increase the perceived threat.The second session for the administrative staff:The trainer delivered a speech about the correct use of personal protective equipment, personal protective equipment required by each department, infection control (disinfection of instruments, equipment, and desktop), emptying the work table and removing common public equipment.	
				Some discussions were held about the issues raised to increase the perceived efficiency. Training on the maintaining and importance of observing social distancing and action in emergencies through role playing. The administrative staff was advised of the consequences of not following the said points.	

### Step 5: Specifying Implementation

In this step, IM helped planners to adopt and implement the programs. The executive team included the head of the dental school. The executives of the program included the middle managers (head of public affairs, administrative, and financial deputy), ward managers, infection control team of the school, and the representatives of SHS2020 standard.

Table 6 illustrates the performance of objectives for the adoption, implementation, and survival of the program. Persuasive communication and discussion were used as effective methods for adopting and implementing the intervention. For this reason, in the first step, a briefing session was held with the program executives, and then they were informed about the objectives and all the program information.

**Table 6:** The matrix of change objectives for teacher and students.

**Determinants**			
**Performance objectives**	**Knowledge**	**Expectations outcome**	**Attitudes**
**Dean of the school**			
PO1- The dean of the school should be familiar with the objectives of the program.	K.1- They should know the program is available. K.2- They should explain that the program is meant for promoting COVID-19 prevention behaviors in the school.	OE1.1- They should expect that if they use this program, it will have educational benefits for students.OE1.2- They should expect that if they use this program, the health of employees, students, professors and patients will be ensured.	A.1.1- They should explain that the coronavirus epidemic is a real and important problem. A.1.2- They should explain that the dental school is one of the most dangerous environments in terms of transmission and spread of the virus. A.1.3- They should explain that this program is easy to run (consider the program relevant current services). A.1.4- They should explain that the implementation of this program is low cost (consider the program relevant current services). A.1.5- They should explain that the implementation of this program does not increase the workload of faculty staff (consider the program relevant current services). A.1.6- They should explain how the program can be improved from what it is now, relate the program to current services, the program should not be too complicated.
PO2- The dean should develop and support the program.PO2.1- The dean should receive the consent of the staff to implement the program.	k.2- They should describe ways to engage staff	OE2.1- They should expect employees to accept the program; if they are given the opportunity to talk about the program, needs and resources, program goals, and organizational support.OE2.2- They should expect employees to feel that they are doing a good job for keeping themselves, their families, students and patients healthy by implementing this program.	A.1- They should express the importance of engaging employees to implement the intervention.
PO3- The dean of the school should make the program run routinely in the organization.PO3.1- They should lay down rules on how students, professors, and medical staff should be present in departments, and on the covering of personal protective equipment, infection control, and treatment protocols.PO3.2They should consider rewards and punishments for following the rules		OE3.1- They should expect that the setting rules would help the program continue to be used.OE3.2- They should expect that considering rewards and punishments for following the rules would help the program continues to be used.	A.3.1- They should explain the importance of finding a way to integrate an intervention program into routine programs. A.3.2- They should explain the importance of setting rules for integrating intervention program into routine programs. A.3.3- They should explain the importance of setting rules for integrating intervention programs into routine programs. A.3.3- They should explain the importance of reward and punishment for integrating the intervention program into routine programs.
**Program executives**			
PO.1- Executives of the program should be loyal to the strategies and methods considered.	K.1- Executives of each department should know their duty in implementing the intervention. K.2- They should explain the protocols notified to themselves for the intervention.	OE.1- They should expect to achieve program goals by following the program steps carefully.	A.1- Executives should explain that it is important to accurately execute program strategies.
PO2.2- Executives to run the program fully.PO2.3- Executives should deliver the program to all target groups.	k.2.1- Executives should name the steps of implementing the intervention. k.2.2- Executives should name the target groups covered by them.	OE.2.1- They should expect to achieve the goals of the program with the full implementation of the program.OE.2.2- They should expect to achieve the goals of the program by implementing interventions designed for all target groups.	A.2.1-Executives should explain that it is important to accurately execute program strategies. A.2.2- Executives should explain that it is important to accurately execute program strategies.

Then, the executive team was informed about the methods and strategies for implementation of the intervention in different target groups and the tasks of the designated teams in detail.

The instructions of the Ministry of Health were also provided for the executors.

### Step 6: Generating Evaluation Plan

Context, Input, Process, and Product evaluation checklist was used to evaluate the program (16). This checklist is composed of 10 components, such as the contract agreements, context, input, process, impact, effectiveness, sustainability, transportability evaluation, Meta evaluation, and final synthesis report (16). To perform the evaluation of the program according to the CIPP model, the activities required for the evaluation were extracted from the last published checklist of the CIPP model (2007). Afterward, with the help of SHS2020 standard representatives, these activities were coordinated with the objectives of SHS2020 standards, and finally, the evaluation checklist was provided and completed during the internal audit.

Context, Input, Process, and Product components were selectively used in different sequences and generally at the same time depending on the need for specific evaluations (16). In this study, eight components, such as contractual agreements, context, input, process, impact, effectiveness, sustainability, the final synthesis report, were applied.

Evaluation in this study encompassed the following steps:

1. Contractual agreements: A clear understanding of the evaluation that needs to be conducted was established.

2. Assessing the conditions and programming context: The needs, strengths and weaknesses, threats, opportunities, and problems in the faculty environment were assessed and evaluated. This step was consistent with the needs assessment phase and performed concurrently with this phase.

3. Evaluation of the program inputs: Existing strategies and programs, capacity and feasibility of the designed program, evaluation of budget and equipment, tools, and materials required to conduct the program were assessed and evaluated. This step was also performed with the needs assessment phase.

4. Evaluation of the program processes: Monitoring, documentation, and evaluation of the program activities were performed, and representatives of SHS2020 standard were involved in monitoring, observation, preserving photographic records, and providing periodic progress reports on the program implementation. At this stage, the checklists were prepared and completed by the standard representatives through observation. By photography and preparing a report regarding the documentation and intervention processes and the periodic progress reports to the faculty officials, they were informed of the program process. This time, it was determined whether all the modules of the program have been implemented? Does the program run according to the predetermined sequences and strategies? Did all the target groups receive the program? To what extent are the targets groups have involved in the program? Is the predetermined performance objective accomplished?

The satisfaction of the target groups related to the intervention was also questioned.

5. Evaluation of the program consequences: The information about the acceptance, modification, or rejection of the program was evaluated by asking from the target groups. The outcomes of this program included: increasing knowledge, perceived threat, perceived efficiency, reducing barriers to understanding, and increasing preventive behaviors against COVID-19 infection, which is obtained through completing the PMT questionnaire by staff, faculty, and students before and after the intervention.

6. Evaluation of the effectiveness of program: The results of the quality and importance of the program must be evaluated. In this regard, the effectiveness of the interventions will be evaluated by the number of involved cases in dental school related to therapeutic and educational processes 3 months after the intervention. After performing above six evaluation steps by the representatives of BRSM Company and providing a report on the observance of applicable professional standards to the faculty officials, the identified defects were fixed. Moreover, the head office of the BRSM Company was invited to refer to the faculty for the final evaluation of the program. After conducting the final evaluations by the faculty, the evaluators of this company, on 28 June, 2020, considered the faculty to be eligible for the SHS2020 standard certification (23).

7. Sustainability evaluation: This stage that will be carried out by BRSM Company 6 months after the initial evaluation, it will be determined to what extent the program has been successfully institutionalized and will continue over time.

8. The final synthesis report: Consistent reports of evaluation findings were collected and communicated to the faculty stakeholders. Reports on the program background, implementation, and results of the program were presented to a wide range of audiences, and they were informed of the interventions carried out and the results of the evaluation by BRSM.

**Figure 2:**
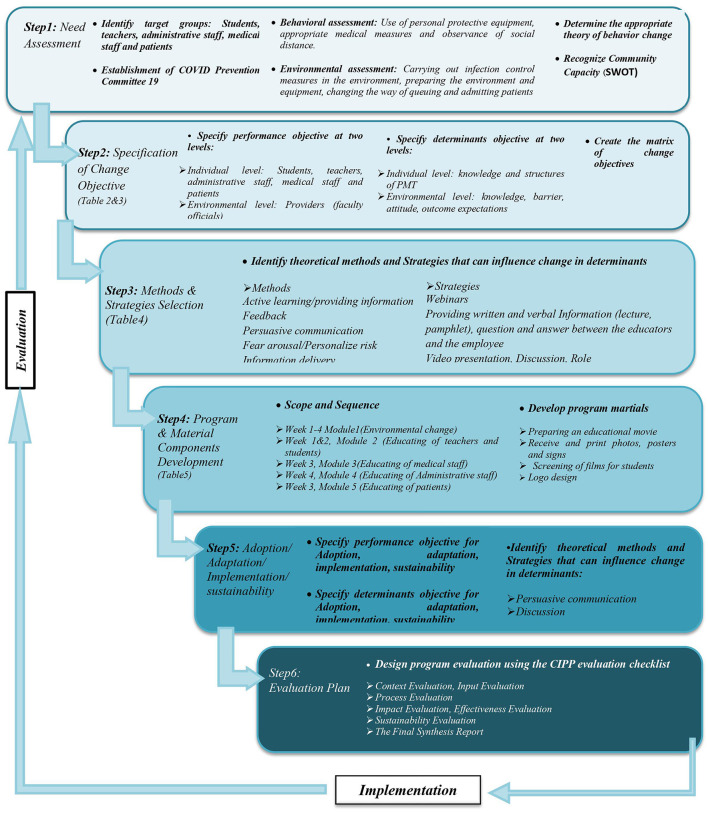
Implementation intervention mapping process.

Figure 2 describes a stepwise process for dental school management during the COVID-19 pandemic based on IM. Further results on the intervention evaluation will be described in a different article.

## Discussion

The present study describes the process design, content, and evaluation of COVID-19 preventive intervention in dental schools. This study for the first time provides practical and simple guidelines for reopening dental schools and providing dental services during the COVID-19 epidemic. Due to the lack of a global protocol for dental services during this pandemic, dental services have been partially or completely disrupted in many countries. In this regard, the lack of standard protocols can increase the prevalence of COVID-19 in dental health centers.

While some national health institutions, such as the NHS, have provided guidance and advice for managing clinical dental emergencies during epidemics (24). This intervention focuses on the behavioral and environmental changes simultaneously. These changes are very important to control and prevent the COVID-19 infection among dentists. In this regard, the present study mainly focused on the identification of COVID-19 preventive behaviors in target groups, factors affecting these behaviors, and determining environmental conditions.

In this study, IM was used as a conceptual framework for the intervention design. Although the design of the intervention was a time-consuming process, it can lead to the implementation of the intervention with a systematic and evidence-based approach.

The Centers for Disease Control and Prevention, ADA, and WHO provided some recommendations for the control of the prevalence of COVID-19 for dentists and dental staff, such as patient screening, infection control (environmental disinfection, equipment and tools, and unit isolation), behavioral measures (use of personal protective equipment and handwashing), and treatment measures (performing essential and emergency treatments, using rubber dam placement, reducing aerosol production during examination and treatment, etc.) (9–11).

The first essential step for the safe management of patients during the pandemic is investigating risk level of patient through screening patients to cut the transmission chain and evaluating the chief complaint of the patients (25). These cases should be managed remotely to avoid useless direct contact with patients that can increase the risk of infection for patients and dentists. In this intervention, to reduce the presence of patients, students, and professors in the school, contact-less treatment and remote learning were performed. These interventions were performed by using a telephone or online appointment system, teledentistry, implementation of virtual teaching, phone or online screening of patients, and installation of PACS. Previous studies have demonstrated that teledentistry (26, 27), virtual teaching (28–30), and PACS software (31) reduced the prevalence of COVID-19 among dental schools through the reduction of contact between individuals.

The most important measures to control the infection included selecting one person to supply the disinfectants for surfaces, equipment, and tools. At the beginning of each day, this person prepared the appropriate disinfectants for the surfaces, equipment, and tools according to the standard protocol and provided them for the medical staff in different wards. Isolation of well-ventilated dental chair unit (with conventional and novel air conditioning systems, such as photocatalytic technologies and windows) was conducted as another important measure of infection control (32).

Fear arousal strategy as behaviors that protect people against life-threatening conditions (such as COVID-19) is often used as an effective way to promote health, prevent disease, and adopt healthy behaviors in society (33). However, this strategy can be effective when people are confident that can perform the recommended measures (self-efficacy), and meeting the recommended measures has favorable consequences (response efficiency) for them. In this study, the PMT model was used to identify the determinants affecting behavioral factors, and methods and strategies of behavioral changes. Regular monitoring of certain behaviors (such as wearing personal protective equipment and medical measures) and rewards and punishment for not following the behaviors as important behavioral measures were considered to increase the effectiveness of the target groups. In this intervention, to avoid overcrowding of people in the faculty and the risks associated with infection, virtual online and offline teaching, webinar lecture, and training sessions were held for the students and professors. Virtual teaching as a suitable method offers many advantages, such as saving time and better performance of students (34).

Staff training was also conducted in small groups in large spaces with proper ventilation.

In the present study, the intervention was designed based on the latest scientific findings in the field of COVID-19 as another important measure. Therefore, a scientific research team was formed to access the latest scientific findings through searching and then inform the planning team every 72 h. The planning team examines the received information scientifically, evaluates their compliance with the facilities and policies of the organization, makes the necessary changes in the program, and informs the project implementers. However, finding a balance between previous and new findings and constantly updating the information regarding the transmission and prevention ways of COVID-19 are still challenges, especially in dentistry.

This study had some limitations at the time of implementation of the intervention. These limitations were related to insufficient scientific information about the nature of the virus like its transmission modes, and incubation time, and due to constant updating information, the effectiveness of the preventive methods remains a challenge.

## Conclusions

This study describes the performance of the Iranian School of Dentistry in developing disease prevention strategies during the COVID-19 pandemic based on the IM process. Our findings indicated that the intervention in dental settings based on the IM process leads to a comprehensive and structured program in dental schools. Therefore, the teamwork can consider the necessary changes in the running programs as the global and national guidelines for the epidemic change. Therefore, it can be concluded that intervention in dental environments based on the IM process can develop a comprehensive and structured program in the dental school.

## Data Availability Statement

The original contributions presented in the study are included in the article/supplementary material, further inquiries can be directed to the corresponding authors.

## Ethics Statement

The studies involving human participants were reviewed and approved by the Hamadan University of Medical Sciences Ethics Committee (approval code: IR.UMSHA.REC.1399.416). This study and the method of obtaining consent were approved by the Ethics Committee. The questionnaire was anonymous and informed consent was considered as agreeing to participate in the survey. Participation in the study was voluntary. The patients/participants provided their written informed consent to participate in this study.

## Author Contributions

AM, SS, SB, NR, AH, MM, HK, and OA contributed to the study concept and design. SS, NR, BB, OA, and AP contributed to the acquisition of data. SS, MM, and AP contributed to the initial drafting of the manuscript. SS, AM, AH, and SB contributed to study supervision. All authors contributed to the interpretation of data and critical revision of the report.

## Funding

We acknowledge funding from the Hamadan University of Medical Sciences (9905283448).

## Author Disclaimer

The content is solely the responsibility of the authors.

## Conflict of Interest

The authors declare that the research was conducted in the absence of any commercial or financial relationships that could be construed as a potential conflict of interest.

## Publisher's Note

All claims expressed in this article are solely those of the authors and do not necessarily represent those of their affiliated organizations, or those of the publisher, the editors and the reviewers. Any product that may be evaluated in this article, or claim that may be made by its manufacturer, is not guaranteed or endorsed by the publisher.

## Abbreviations

COVID-19: Coronavirus disease 2019
IM: Intervention Mapping
PMT: Protection Motivation Theory
SARS-CoV-2: Severe acute respiratory syndrome coronavirus 2
CDC: Disease Control and Prevention
ADA: American Dental Association
WHO: World Health Organization
CPDS: COVID-19 Prevention Committee at the School of Dentistry
OSHA: Occupational Safety and Health Administration
SHS: Safety and Hygiene Scheme standard
NHS: National Health Service
CIPP: Context, Input, process, product
PPE: Personal protective equipment.
